# The role of ultrasound in predicting non-invasive ventilation outcomes: a systematic review

**DOI:** 10.3389/fmed.2023.1233518

**Published:** 2023-10-31

**Authors:** Matthew Kheir, Vincent Dong, Victoria Roselli, Bushra Mina

**Affiliations:** ^1^Division of Pulmonary and Critical Care Medicine, Department of Medicine, Lenox Hill Hospital - Northwell Health, New York, NY, United States; ^2^Donald and Barbara Zucker School of Medicine at Hofstra/Northwell, Hempstead, NY, United States; ^3^Department of Medicine, Lenox Hill Hospital - Northwell Health, New York, NY, United States; ^4^Office of Clinical Research, The Feinstein Institutes for Medical Research, Northwell Health, Manhasset, NY, United States

**Keywords:** non-invasive, ventilation, outcomes, lung, ultrasound, diaphragm, dysfunction

## Abstract

**Purpose:**

To systematically review and compare ultrasonographic methods and their utility in predicting non-invasive ventilation (NIV) outcomes.

**Methods:**

A systematic review was performed using the PubMed, Medline, Embase, and Cochrane databases from January 2015 to March 2023. The search terms included the following: ultrasound, diaphragm, lung, prediction, non-invasive, ventilation, and outcomes. The inclusion criteria were prospective cohort studies on adult patients requiring non-invasive ventilation in the emergency department or inpatient setting.

**Results:**

Fifteen studies were analyzed, which comprised of 1,307 patients (*n* = 942 for lung ultrasound score studies; *n* = 365 patients for diaphragm dysfunction studies). Lung ultrasound scores (LUS) greater than 18 were associated with NIV failure with a sensitivity 62–90.5% and specificity 60–91.9%. Similarly, a diaphragm thickening fraction (DTF) of less than 20% was also associated with NIV failure with a sensitivity 80–84.6% and specificity 76.3–91.5%.

**Conclusion:**

Predicting NIV failure can be difficult by routine initial clinical impression and diagnostic work up. This systematic review emphasizes the importance of using lung and diaphragm ultrasound, in particular the lung ultrasound score and diaphragm thickening fraction respectively, to accurately predict NIV failure, including the need for ICU-level of care, requiring invasive mechanical ventilation, and resulting in higher rates of mortality.

## Introduction

There are several contributors in predicting the outcome of non-invasive ventilation (NIV). Historically, multiple articles have demonstrated that the prediction of noninvasive ventilation (NIV) failure was based on components of the patient’s vital signs, level of consciousness, and degree of acidosis (1–6). This led to the development of a scale that considers heart rate, acidosis, consciousness, oxygenation, and respiratory rate (referred to as the HACOR scale) and is used to predict NIV failure, defined as the need for intubation after NIV intervention (7, 8). The scale was however limited in terms of predictive value in respiratory illnesses and thus an updated HACOR score was developed that considered baseline data such as acute respiratory distress syndrome (ARDS), septic shock, immunosuppression, organ failure, among other data (9). Additional risk factors beyond the HACOR scale for NIV failure include patient baseline severity scores (SOFA, APACHE II and SAPS II), delay between admission and NIV use, duration of NIV use, patient-ventilator asynchrony, number of fiberoptic bronchoscopies performed, and increased radiographic infiltrates within the first 24 hours (5, 10–13).

Point of care ultrasound (POCUS) in the fields of pulmonary and critical care medicine has received increased attention because of its rapid availability to assess and diagnose patients, accuracy, reproducibility, low-cost profile, and lack of harmful radiation. Numerous studies have attempted to utilize various ultrasonographic methods to ultimately predict NIV outcomes. However, given the variability of each technique and the multiple sites of interest for bedside ultrasound, it is challenging to comprehend the clinical significance and the generalized acceptance of specific ultrasound modalities.

The purpose of this study is to systematically review the literature to highlight the predictiveness of bedside ultrasound with regards to NIV outcomes as well as to compare the various ultrasonographic techniques that have been used.

## Materials and methods

This study was conducted in accordance with the 2020 PRISMA (Preferred Reporting Items for Systematic Reviews and Meta-Analyses) statement and the PRISMA checklist (14). A systematic review of the literature from 2015 to 2023 on the role of ultrasound in predicting NIV outcomes was performed across a 4-database wide search including PubMed, Medline, Embase, and Cochrane. The queries were performed in March 2023 with the following search terms: ultrasound, diaphragm, lung, prediction, non-invasive, ventilation, and outcomes.

### Inclusion/exclusion criteria

The inclusion criteria were adults 18 years of age and older, patients in the emergency department or hospitalized, with acute respiratory failure, receiving ultrasound assessments, and on non-invasive ventilation. The exclusion criteria were studies in languages other than English, pregnant patients, post-surgical patients, patients with neuromuscular disorders, using ultrasound to determine if patient needs to be advanced to NIV, using ultrasound to evaluate if patient can be extubated or weaned from invasive mechanical ventilation, and if the study involved drug interventions. Two investigators (M.K. and V.D.) reviewed the titles and abstracts of all articles that met the inclusion and exclusion criteria, and full-text articles were obtained to verify that the criteria were met.

## Results

**Figure 1:**
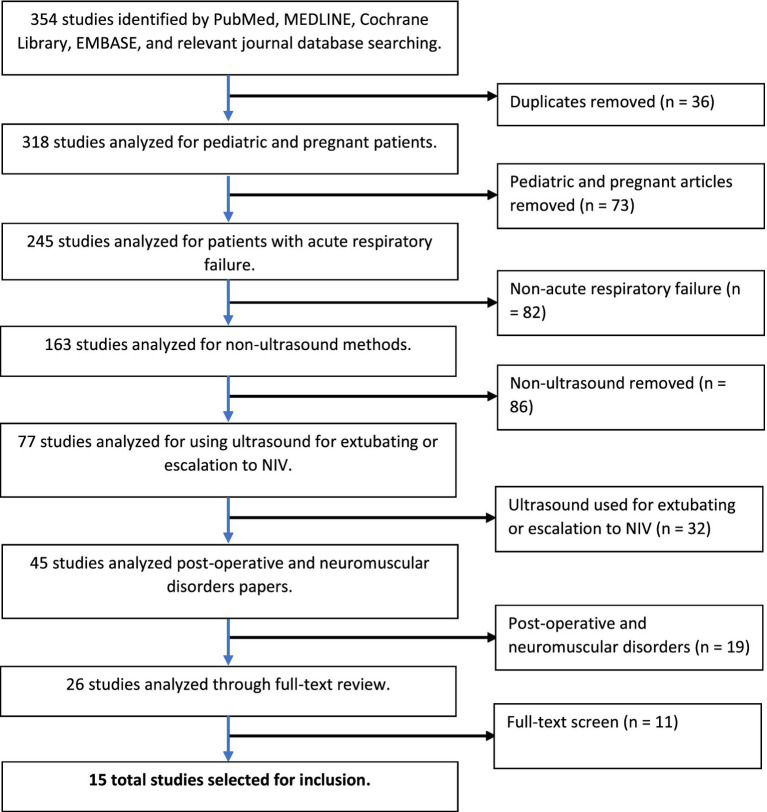
PRISMA (preferred reporting items for systematic reviews and meta-analyses) flowchart of literature search.

A flow diagram documenting the method of article identification and selection is shown in Figure 1. A total of 318 studies remained after eliminating 36 duplicate studies. The investigators independently reviewed the titles and abstracts, identifying 26 full-text articles for review. Application of the inclusion and exclusion criteria yielding 15 full-text articles for inclusion in the review (15–29).

### Lung ultrasound score

**Figure 2:**
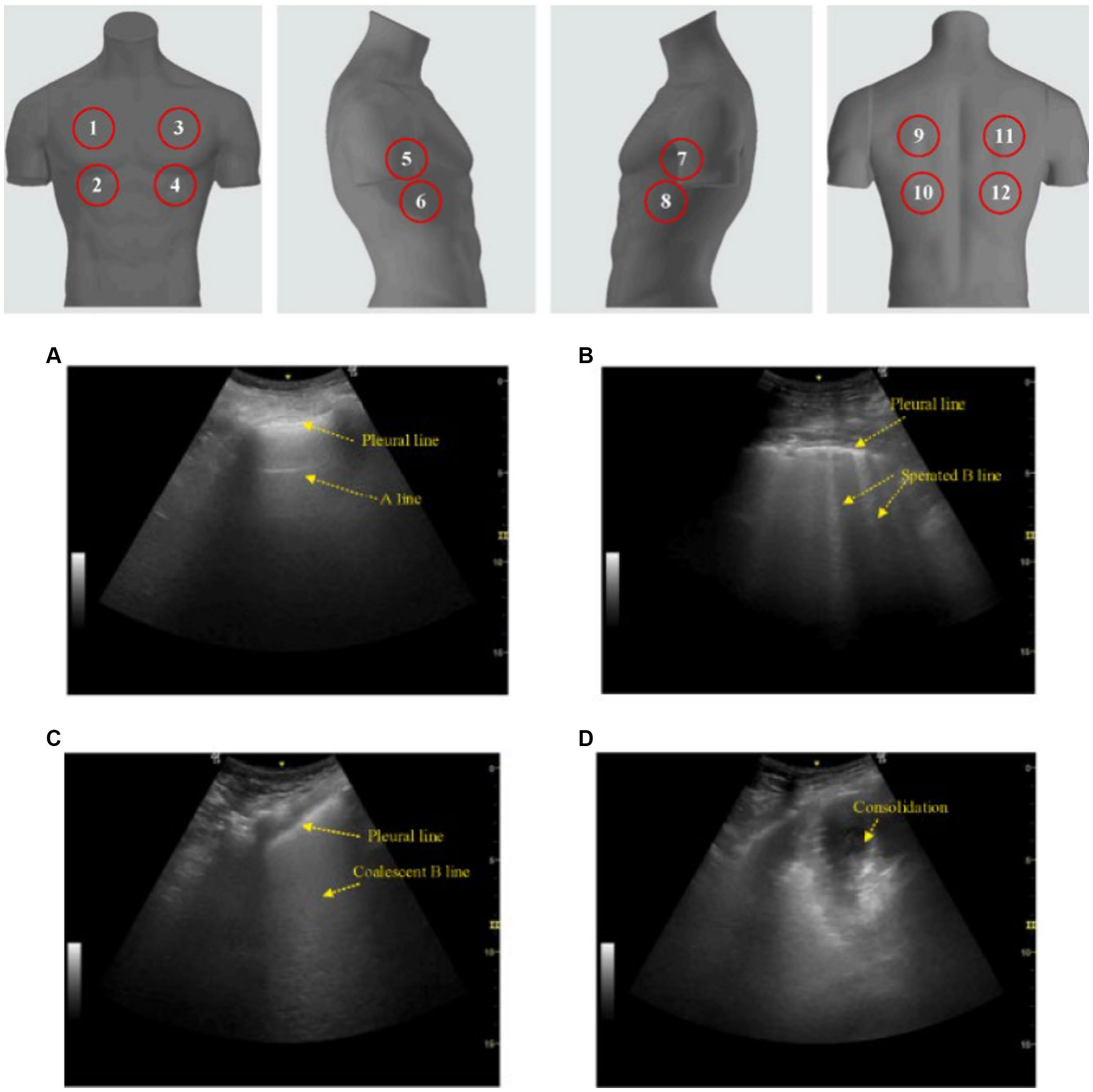
Lung ultrasound score (LUS). The classic LUS requires scanning 12 zones (upper image; 6 on each hemithorax) while scoring each zone from 0 to 3 based on pattern. **(A)** A score of 0 is a normal pattern. **(B)** A score of 1 requires at minimum 3 isolated or coalescent B-lines covering <50% of the screen without clear subpleural alterations. **(C)** A score of 2 is given when B-lines encompass >50% of the screen without clear subpleural alterations. **(D)** And finally, a score of 3 is made when consolidation is observed. Total scores range from 0 to 36. This image is reproduced with permission from the Elsevier Novel Coronavirus Center. No changes were made to this image (38).

Several studies employ an ultrasonographic method of scoring lung pathology, namely the lung ultrasound score (LUS) (Table 1). A lung ultrasound score assessment is performed for multiple reasons, such as: perioperative oxygenation (31), predicting disease severity and mortality in ARDS (32), monitoring of lung aeration changes (after proning in ARDS, antimicrobial therapy in ventilator-associated pneumonia, selecting the ideal positive end-expiratory pressure, etc.) (33–35), weaning from mechanical ventilation (36), and as a prognosticator for respiratory failure (15, 18–20, 22, 28). There are multiple variations of the LUS, however arguably the more commonly utilized version of the LUS assessment consists of scanning a predetermined 6 regions per lung. Lung aeration of each region is graded between 0 to 3 depending on the ultrasound pattern visualized. In each region, points are allocated according to the following ultrasound pattern: normal = 0, well-defined B-lines = 1, coalescent B-lines = 2, and consolidation = 3. The total score for the LUS assessment therefore ranges from 0 to 36 (37) (Figure 2). A broader score, known as the integrated lung ultrasound score (i-LUS), was implemented during the coronavirus disease 2019 (COVID-19) pandemic that incorporates other important parameters seen with COVID-19 pneumonia. These additional parameters include: presence of pleural effusion (absent = 0, present = 1), presence of pericardial effusion (absent = 0, present = 1), measurement of IVC respiratory variation (<0–33%; absent = 0, present = 1), and diaphragm excursion (excursion >2 ± 0.5 cm was considered normal = 0, values below = 1) (18). Again, there are several other variations of the standard LUS (most of which are described in this present article) and these lung ultrasound score assessments have demonstrated positively in predicting outcomes for patients on NIV.

**Table 1:** Characteristics and findings for articles utilizing lung ultrasound score.

Study/year	Country	Design/setting/population	Variable	Number of patients	Age (mean, standard deviation)	Male sex, %	Findings
Ji et al. (19), 2020	China	Prospective/Hospitalized/COVID-19 Pneumonia	LUS	280	55 (n.a.)	50.4	Patients with a high LUS score (LUS > 12) were more likely to require invasive mechanical ventilation, had higher incidence of ARDS, and had higher mortality compared to the lowest LUS score group (LUS 0–1).
Lichter et al. (30), 2020	Israel	Prospective/Hospitalized/COVID-19 Pneumonia	LUS	120	64.7 ± 18	62	Optimal cutoff for LUS score was 18 (Sn 62% and Sp 74%), for which both mortality and requirement for invasive mechanical ventilation were increased. Hazard ratio of IMV or death was 1.12 per LUS point.
Biasucci et al. (28), 2021	Italy	Prospective/ED/COVID-19 Pneumonia	LUSsc and LUSq	85	64 ± 14	71.8	LUSsc and LUSq were significantly higher in patients who failed NIV than those who did not.
Ahmed et al. (15), 2022	Egypt	Prospective/Hospitalized/ ARF	LUS	50	59 ± 13	41.4	LUS cut-off value of 18 on admission showed Sn 77.0% and Sp 60.0% of predicting NIV failure. LUS cut-off value of 15 after 12 h of NIV use showed Sn 87.0% and Sp 75.0%.
Blair et al. (22), 2022	USA	Prospective/ED and Hospitalized/COVID-19 Pneumonia	mLUS	264	61 (n.a.)	56.8	Every incremental increase in mLUS score at enrollment was associated with an increased disease progression to ICU-level of care as well as 28-day mortality (adjusted hazard ratio of 3.61 and 3.10, respectively).
Dell’Aquila et al. (18), 2022	Italy	Prospective/ED/COVID-19 Pneumonia	i-LUS	143	71.5 ± 14.9	59.4	Survivor group median i-LUS score of 16, while the non-survivor group score was 20 in the non-survivor group.

LUS = lung ultrasound score (12 scanning zones; ranges from 0 to 36), LUSsc = lung ultrasound score (6 scanning zones; ranges from 0 to 18), LUSq = number of involved zones on lung ultrasound (ranges from 0 to 6), mLUS = mean lung ultrasound score (12 scanning zones; ranges from 0 to 3), i-LUS (LUS score with 4 additional parameters: pleural effusion, pericardial effusion, IVC respiratory variation, and diaphragm excursion), ED, emergency department; ARF, acute respiratory failure; ARDS, acute respiratory distress syndrome; NIV, non-invasive ventilation; IMV, invasive mechanical ventilation; ICU, intensive care unit; Sn, Sensitivity; Sp, specificity.

Ahmed et al. performed a prospective observational study of 50 ICU patients who presented with acute respiratory failure and were indicated for NIV (including COPD with exacerbation, pneumonia with no or mild secretions, acute lung injury manifested by PaO2/FiO2 < 200, and acute congestive heart failure with pulmonary edema). Lung ultrasound score, HACOR score, and serum lactate were recorded on admission and after 12 h from NIV application. To predict NIV failure, LUS with a cut-off value of 18 on admission showed sensitivity 77.0%, specificity 60.0% (*p* = 0.001). A cut-off value of 15 after 12 h NIV use showed a sensitivity of 87.0% and a specificity of 75.0% (*p* < 0.001) (15).

A similar study of 120 patients admitted for COVID-19 pneumonia showed that the optimal cutoff for the baseline LUS score was 18 (sensitivity 62%, specificity 74%). Both mortality and requirement for invasive mechanical ventilation were increased with a baseline LUS score > 18 compared to patients with baseline LUS score between 0 and 18. The hazard ratio of invasive mechanical ventilation or death for LUS score was 1.12 per point (*p* = 0.0008) (30). Dell’Aquila et al. also examined COVID-19 patients in a prospective study of 143 patients. They noted that in the survivor group, patients had a median i-LUS score of 16, while the score was 20 in the non-survivor group (interquartile range 12–20 vs. 15–24; *p* = 0.005) (18).

In a prospective cohort study of 85 patients with COVID-19 respiratory failure, Biasucci et al. showed that LUS (denoted as “LUSsc”; median score of 12 for NIV failure and median score of 6 for NIC success) as well as the amount of involved sonographic lung areas (denoted as “LUSq”; median areas involved were 6 for NIV failure and median areas involved were 3 for NIC success) were significantly higher in patients who failed NIV (*p* < 0.001) (28). This was also seen in a larger study of 280 patients with COVID-19 pneumonia by Ji et al., which demonstrated that patients with a high LUS score (defined as LUS > 12) were more likely to have poor outcomes including requiring invasive mechanical ventilation, higher incidence of ARDS, and higher mortality rates compared to the lowest LUS score group (defined as LUS 0–1), with a specificity and sensitivity of 90.5 and 91.9%, respectively (19). Blair et al. utilized the mean LUS (mLUS) score (ranging from 0 to 3) across 12 lung zones. The study revealed that every increase in mLUS score at enrollment was associated with disease progression to ICU-level of care (adjusted hazard ratio [aHR], 3.61; 95% confidence interval [CI], 1.27–10.2; *p* = 0.016) as well as 28-day mortality (aHR, 3.10; 95% CI, 1.29–7.50; *p* = 0.012) (22).

### Diaphragm dysfunction

**Figure 3:**
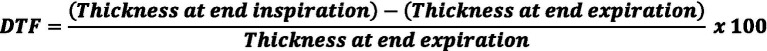
Equation for diaphragm thickness fraction (DTF).

**Figure 4:**
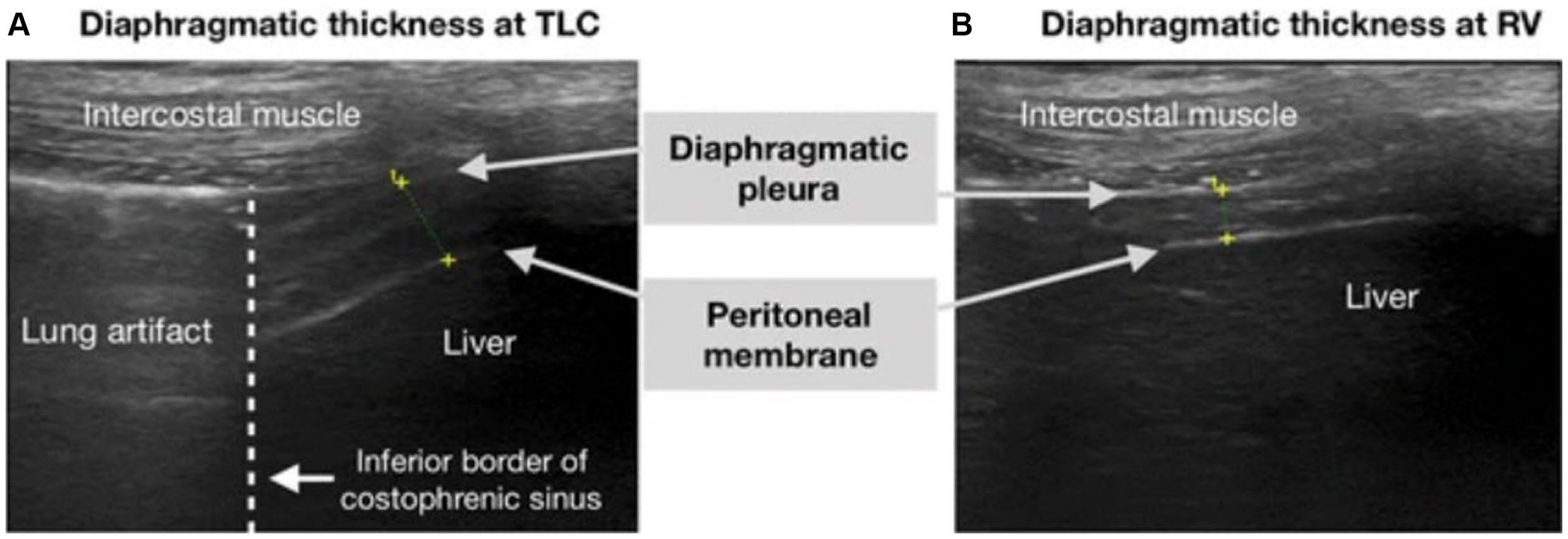
Obtaining diaphragm thickness fraction on ultrasound. Ultrasound image of the diaphragm on B-mode, showing the three layers of the diaphragm (hypoechoic diaphragm bordered by hyperechoic diaphragmatic pleura and the peritoneal membrane). Seen in **(A)** total lung capacity (TLC) and **(B)** residual volume, which is equivalent to end-inspiration and end-expiration, respectively. This image was reproduced with the permission of the Creative Commons license. No changes were made to this image. The Creative Commons Public Domain Dedication waiver (http://creativecommons.org/publicdomain/zero/1.0/) applies to this image (41).

In addition to the lung ultrasound score, point of care ultrasonography can be used to rapidly assess for many pathologies including diaphragm dysfunction (DD) (Table 2). Similar to other ultrasound modalities, diaphragm-related ultrasound is noninvasive, safe, and can serve as a repeatable bedside tool. Patients with significant hypoxia who require NIV have a risk of developing diaphragmatic impairment which may poorly affect outcomes, including the need for invasive mechanical ventilation. Numerous studies have employed ultrasonography to assess functionality of the diaphragm to assist in predicting outcomes for patients on NIV (16, 17, 21, 23–27). These studies assessed diaphragm thickness (DT) or the variation of diaphragmatic thickness fraction (DTF) between end-inspiration and end-expiration. DTF is calculated from the following formula: [(Thickness at end-inspiration − Thickness at end-expiration)/(Thickness at end expiration)] × 100, see Figure 3. A DTF, also known as change in diaphragmatic thickness (ΔTdi), <20% is consistent with diaphragmatic dysfunction (39) (Figure 4). For sake of consistency, this article will use the term DTF with knowledge that it is interchangeable with ΔTdi.

**Table 2:** Characteristics and findings for articles utilizing diaphragm dysfunction.

Study/year	Country	Design/setting/population	Variable	Number of patients	Age (mean, standard deviation)	Male sex, %	Findings
Antenora et al. (21), 2016	Italy	Prospective/Hospitalized/ AECOPD	DTF	41	76 (n.a.)	63.4	The presence of DTF < 20% on admission was associated with NIV failure, increased stay in ICU, prolonged use of IMV and a higher mortality rate.
Marchioni et al. (17), 2018	Italy	Prospective/Hospitalized/AECOPD	DTF	75	78 (n.a.)	51	Patients with a DTF < 20% have significantly higher risk of NIV failure and mortality (risk ratio = 4.4).
Cammarota et al. (27), 2019	Italy	Prospective/ED/AECOPD	DT and DE	22	Median age 79	38	Diaphragmatic excursion assessment 2 h after NIV initiation was a better predictor of NIV failure than left expiratory diaphragmatic thickness, pH, and paCO2 (NIV success 1.99 cm vs. NIV failure 1.20 cm excursion).
Corradi et al. (23), 2020	Italy	Prospective/Hospitalized/COVID-19 Pneumonia	DTF	27	Median age 66	85	NIV failure significantly associated with low DTF. The best DTF threshold for predicting NIV failure was 21.4% with Sn 94.4% and Sp 88.9%.
Barbariol et al. (29), 2021	Italy	Prospective/Hospitalized/De-novo ARF	DE	47	65.5 ± 14.8	57.4	NIV failure associated with low DE (not statistically significant). As a predictor for NIV outcomes, DE had an AUC 0.53 with a Sn 58.1% and Sp 62.5%.
Corradi et al. (23), 2021	Italy	Prospective/Hospitalized/COVID-19 Pneumonia	DT	77	Median age 59	66.2	Individuals who developed adverse outcomes on NIV had thinner diaphragms than those who did not (2.0 vs. 2.2 mm).
Kocyigit et al. (24), 2021	Turkey	Prospective/ED/AECOPD	DTF	60	70.9 ± 8.8	78.3	DTF < 20% has Sn 84.6% and Sp 91.5% in predicting NIV failure.
Mercurio et al. (26), 2021	Italy	Prospective/ED/De-novo ARF	DTF	18	66 ± 19	44.5	DTF < 36.3% significantly predicted NIV failure with Sn 71.7% and Sp 94.3%.
Elsayed et al. (16), 2022	Egypt	Prospective/Hospitalized/AECOPD	DTF	75	59.3 ± 10.1	85.3	DTF < 26–29% on both sides (left hemidiaphragm% - right hemidiaphragm%) had Sn 96.67% and Sp 80–82.22% in predicting NIV failure.

DT, diaphragm thickness; DE, diaphragmatic excursion; DTF, diaphragm thickness fraction; DTF formula, [(Thickness at end-inspiration − Thickness at end-expiration)/(Thickness at end expiration)] × 100; AECOPD, acute exacerbation of COPD; ED, emergency department; ARF, acute respiratory failure; NIV, non-invasive ventilation; IMV, invasive mechanical ventilation; ICU, intensive care unit; Sn, Sensitivity; Sp, specificity.

**Figure 5:**
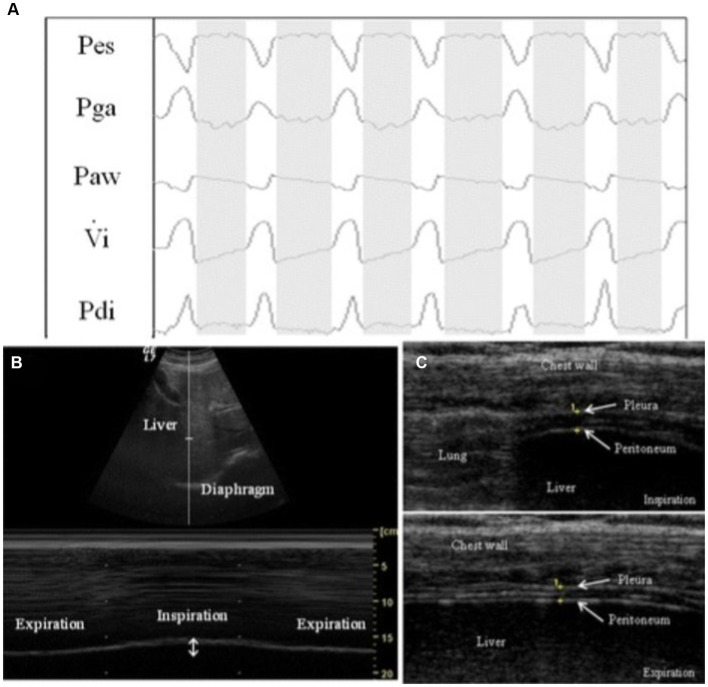
Example of respiratory tracings with ultrasound measurements during a pressure support of 0 cm H_2_O (PS_0_ step). **(A)** Respiratory tracings showing: Pes, esophageal pressure; Pga, gastric pressure; Paw, airway pressure; Vi, respiratory flow; Pdi, transdiaphragmatic pressure. The white columns represent inspiration and gray columns represent expiration phases. **(B)** Ultrasound view of diaphragm excursion during spontaneous breathing in B-mode (upper) and M-mode (lower). **(C)** Ultrasound view of the diaphragm in the zone of apposition during inspiration (upper) and during expiration (lower). The diaphragm is the three-layer structure in the middle consisting of a hypoechoic central layer sounded by an echogenic diaphragmatic pleurae and peritoneum, as indicated by the yellow arrows. This image was adopted with the permission of the Creative Commons license. No changes were made to this image. The Creative Commons Public Domain Dedication waiver (http://creativecommons.org/publicdomain/zero/1.0/) applies to this image (40).

Across the more recent studies which are highlighted in this article, diaphragm evaluation was performed on patients with an acute exacerbation of COPD (AECOPD) or with acute respiratory failure from SARS-COV2 infection. Marchioni et al. performed a prospective observational study using ultrasound to assess diaphragm dysfunction in patients with AECOPD and reported that patients with diaphragmatic dysfunction had a higher risk for NIV failure than those without DD (risk ratio = 4.4; *p* < 0.001). Change in diaphragm thickness (DTF) <20% during tidal volume was the predefined cutoff for identifying DD. The study investigated the correlation between ultrasound-assessed DD and the transdiaphragmatic pressure (Pdi) assessed using an invasive sniff maneuver [esophageal pressure (Pes) and gastric pressure (Pga) levels are recorded using balloon catheters before starting NIV; transdiaphragmatic generating pressure capacity (Pdi) was obtained by subtracting Pes from Pga (Pga – Pes) during a sniff maneuver], see Figure 5. It was reported that DTF highly correlated and had similar accuracy in identifying diaphragmatic dysfunction as with Pdi sniff (Pearson’s *r* = 0.81; *p* = 0.004). Lastly, the study demonstrated that DTF < 20% had better accuracy in predicting NIV failure than baseline pH value <7.25, as well as early changes in arterial pH and PaCO2 following initiation of NIV (AUCs 0.84, 0.51, 0.56, and 0.54, respectively; *p* < 0.0001) (17).

Cammarota et al. similarly reported diaphragmatic excursion assessment 2 h after NIV initiation was a better predictor of NIV failure than pH, PaCO2, and left expiratory diaphragmatic thickness. Diaphragmatic excursion was greater in NIV successes than in NIV failures at initiation of NIV (1.92 [1.22–2.54] cm vs. 1.00 [0.60–1.41] cm, *p* = 0.02) and at 2 h after initiation of NIV (1.99 [1.63–2.54] cm vs. 1.20 [0.79–1.41] cm, *p* = 0.008), respectively (27). Diaphragmatic dysfunction was also evaluated through diaphragmatic excursion by Barbariol et al. in ICU patients admitted for acute respiratory failure. Data was collected right before starting NIV as well as 1 h after initiating NIV. Diaphragmatic dysfunction was defined as a diaphragmatic excursion of less than 1.00 cm. Out of 47 patients, the patients without diaphragmatic dysfunction had about a 10% increase in NIV success than patients with diaphragmatic dysfunction, which was not statistically significant (*p* = 0.54). They also performed ROC analysis and noted that when using diaphragmatic excursion as a predictor of NIV response the area under the curve was 0.53; the best sensitivity (58.1%) and specificity (62.5%) was obtained with a diaphragmatic excursion cut-off of 1.37 cm (29).

Elsayed et al. conducted a prospective observational study where patients with acute exacerbation of COPD (AECOPD) were categorized into successful and failed NIV groups and DTF was measured. Readings of DTF in the successful NIV group were ≥ 33–38% (*p* < 0.001) while failed NIV had DTF values ≤16–18% (*p* < 0.001). In addition, cut-off value of DTF < 26–29% on both sides (left hemidiaphragm% - right hemidiaphragm%) was associated with NIV failure with 96.67% sensitivity, 80–82.22% specificity, 76.3–78.4% positive predictive value (PPV), and a 97.3–97.4% negative predictive value (NPV) (16). In a similar study by Kocyigit et al. on patients with AECOPD in the emergency department, DD (defined as DTF of <20% during spontaneous breathing) had a high sensitivity of 84.6% (95% CI:54.6–98.1), specificity of 91.5% (95% CI:79.6–97.6), PPV 73.3% (95% CI:51.2–87.8), and NPV 95.6% (95% CI:85.7–98.7) (24). Antenora et al. underwent a pilot study on the prevalence and clinical consequences of diaphragmatic dysfunction diagnosed by ultrasonography during AECOPD and reported that DD (defined as DTF < 20% during spontaneous breathing) was found to be strongly correlated with NIV failure (*p* < 0.001, *R*^2^ = 0.27), longer ICU stay (*p* = 0.02, *R*^2^ = 0.13), prolonged mechanical ventilation (*p* = 0.023, *R*^2^ = 0.15), and need for tracheostomy (*p* = 0.006, *R*^2^ = 0.20) (21). In a study by Mercurio et al. on patients with de-novo acute respiratory failure, cut-off values for DTF were explored that would accurately predict NIV failure. It was determined that the cut-off value of DTF < 36.3% significantly predicted NIV failure (*p* < 0.0001) with sensitivity of 71.7% (95% CI 56.5–84.0) and specificity of 94.3% (95% CI 80.8–99.3) (26).

In SARS-COV2 patients, Corradi et al. underwent a single-center pilot study that reported in their univariate logistic regression analysis that CPAP failure was significantly associated with a low DTF (odds ratio [OR]: 0.673; *p* < 0.001) and high respiratory rate (OR: 1.572; *p* < 0.001), but only DTF reached statistical significance at multivariate analysis (OR: 0.681; *p* < 0.001) (23). The same investigators performed a separate study and noted that patients on NIV who developed adverse outcomes had thinner diaphragms than those who did not (2.0 vs. 2.2 mm, *p* = 0.001) (25).

## Discussion

Predicting NIV failure remains a diagnostic dilemma in general practice. Recent literature shows that patients presenting with acute respiratory failure have an NIV failure rate around 31–50% with nearly 65% of NIV failures occurring within 1–48 h of NIV use (8, 42–44). Current methods of evaluating for impending NIV failure is based on a multitude of parameters, of which some include monitoring vital signs, evaluating for signs of respiratory distress on physical examination, obtaining arterial blood gas values, calculating severity scores (e.g., SOFA, APACHE II, etc.), serial chest radiographs, and so forth (3, 6–9, 13, 43, 45). Undoubtedly these measures are crucial, but ongoing monitoring and the time required to obtain all this information can be tedious and laborious. The main findings of this systematic review emphasize the effective ability of utilizing bedside ultrasound as a potential *independent* predictive tool for NIV outcomes.

This study demonstrates that point of care ultrasound of the lung (e.g., lung ultrasound score) and diaphragm (e.g., diaphragm thickening fraction) can predict NIV failure early on (15–19, 21–30). This is pivotal as a delay in early detection of potential NIV failure has been shown to increase length of hospital stay, delay endotracheal intubation and increase hospital morbidity and mortality (45–48). Also, this may serve as an additional tool in guiding clinicians when considering patient disposition with regard to deciding escalating level of monitoring to an intermediate care unit (i.e., stepdown unit) vs. an intensive care unit. Clinicians may also use these ultrasound tools to communicate with family members and/or healthcare proxies about suspected outcomes for the patient. Additionally, clinicians may use this information to proactively prepare for anticipated outcomes of NIV failure (such as longer ICU stay, prolonged mechanical ventilation, mortality, and need for tracheostomy) by either providing closer patient monitoring or even considering additional treatment modalities. A recent emergency department study evaluating COVID-19 patients showed that LUS improved prognostic stratification over clinical judgment alone and supported standardized disposition decisions (49).

In a more pragmatic way of interpreting these articles, one may suggest based on these findings that perhaps a lung ultrasound score greater than 18 (out of 36 total) in a patient places the individual at a higher risk for NIV failure (e.g., ICU admission, invasive mechanical ventilation, and mortality). Likewise, a low diaphragm thickness fraction (also known as change in diaphragm thickness) accurately predicts diaphragm dysfunction. A corresponding DTF value of less than 20% highly correlates with NIV failure. Diaphragm thickness and diaphragmatic excursion were evaluated in three of the nine diaphragm ultrasound studies that met inclusion criteria (25, 27, 29). The two studies in this review examining diaphragmatic excursion showed mixed results in terms of predicting NIV failure, whereas one of those studies concluded that diaphragm thickness was a better predictor of NIV outcomes compared to diaphragmatic excursion. The inconsistency of the results and lack of studies evaluating these two variables, diaphragm thickness and diaphragmatic excursion, make it difficult to interpret and utilize these ultrasonographic methods clinically. Currently, additional research is required to evaluate these ultrasound techniques in the setting of predicting NIV outcomes.

Although the outcomes of many of these studies are promising and exemplify the independent utility of performing bedside ultrasound to predict NIV failure, there are some limitations. Firstly, all of the aforementioned studies are small unblinded prospective observational studies and many are conducted at a single institution. In addition, a vast majority of these studies focus on acute respiratory failure from either COVID-19 or AECOPD, which decreases generalizability to other patient populations (e.g., congestive heart failure, post-operative, bacterial pneumonia, etc.). Moreover, the timing of the ultrasound evaluation and the treatment course of the patients are not standardized, making the external validity of the results difficult to establish. There were also many lung ultrasound scoring systems (e.g., LUS, mLUS, i-LUS, etc.) and different cut-off values for both LUS and DTF to determine the same outcomes. This heterogeneity makes it difficult to perform a pooled analysis. Finally, there were unfortunately only two studies that fit the inclusion criteria that examined diaphragmatic thickness and two studies that examined diaphragmatic excursion as predictors for NIV failure. Hence, much of the conversation regarding diaphragmatic dysfunction centered on the diaphragm thickening fraction instead.

## Conclusion

The results of this systematic review emphasize the clinical utility of performing bedside ultrasonography to predict non-invasive ventilation outcomes. Lack of early recognition of impending NIV failure in patients can be deleterious as these individuals often require ICU admission, invasive mechanical ventilation, and have higher mortality rates. Both the LUS and DTF (i.e., indicators of lung parenchymal injury and diaphragmatic dysfunction, respectively) have been shown to accurately predict NIV outcomes. Further larger prospective cohort studies are warranted to contribute to standardizing LUS and DTF across a wide range of etiologies of acute respiratory failure. Moreover, additional investigations are necessary to evaluate whether a combination of the LUS, DTF, and HACOR score can best predict NIV outcomes.

## Data availability statement

The original contributions presented in the study are included in the article/supplementary material, further inquiries can be directed to the corresponding author.

## Ethics statement

Ethical approval was not required for the study involving humans in accordance with the local legislation and institutional requirements. Written informed consent to participate in this study was not required from the participants or the participants’ legal guardians/next of kin in accordance with the national legislation and the institutional requirements.

## Author contributions

MK and BM: conceptualization. MK, VD, and BM: methodology and writing – review and editing. MK and VD: investigation, data curation, and original draft preparation. MK and VR: image contributions. BM: visualization and supervision. All authors contributed to the article and approved the submitted version.
